# Antibody to Ricin A Chain Hinders Intracellular Routing of Toxin and Protects Cells Even after Toxin Has Been Internalized

**DOI:** 10.1371/journal.pone.0062417

**Published:** 2013-04-24

**Authors:** Kejing Song, R. Ranney Mize, Luis Marrero, Miriam Corti, Jason M. Kirk, Seth H. Pincus

**Affiliations:** 1 Research Institute for Children, Children’s Hospital, New Orleans, Louisiana, United States of America; 2 Department of Cell Biology and Anatomy, Louisiana State University Health Sciences Center, New Orleans, Louisiana, United States of America; 3 Imaging Core, Louisiana State University Health Sciences Center, New Orleans, Louisiana, United States of America; 4 Departments of Pediatrics and Microbiology, Immunology, and Parasitology, Louisiana State University Health Sciences Center, New Orleans, Louisiana, United States of America; 5 Carl Zeiss Microimaging, Thornwood, New York, United States of America; Institute Pasteur, France

## Abstract

**Background:**

Mechanisms of antibody-mediated neutralization are of much interest. For plant and bacterial A-B toxins, A chain mediates toxicity and B chain binds target cells. It is generally accepted and taught that antibody (Ab) neutralizes by preventing toxin binding to cells. Yet for some toxins, ricin included, anti-A chain Abs afford greater protection than anti-B. The mechanism(s) whereby Abs to the A chain neutralize toxins are not understood.

**Methodology/Principal Findings:**

We use quantitative confocal imaging, neutralization assays, and other techniques to study how anti-A chain Abs function to protect cells. Without Ab, ricin enters cells and penetrates to the endoplasmic reticulum within 15 min. Within 45–60 min, ricin entering and being expelled from cells reaches equilibrium. These results are consistent with previous observations, and support the validity of our novel methodology. The addition of neutralizing Ab causes ricin accumulation at the cell surface, delays internalization, and postpones retrograde transport of ricin. Ab binds ricin for >6hr as they traffic together through the cell. Ab protects cells even when administered hours after exposure.

**Conclusions/Key Findings:**

We demonstrate the dynamic nature of the interaction between the host cell and toxin, and how Ab can alter the balance in favor of the cell. Ab blocks ricin’s entry into cells, hinders its intracellular routing, and can protect even after ricin is present in the target organelle, providing evidence that the major site of neutralization is intracellular. These data add toxins to the list of pathogenic agents that can be neutralized intracellularly and explain the in vivo efficacy of delayed administration of anti-toxin Abs. The results encourage the use of post-exposure passive Ab therapy, and show the importance of the A chain as a target of Abs.

## Introduction

Plant and bacterial protein toxins play a major role in disease pathogenesis and are of biodefense concern. Such toxins generally have a two domain structure, where the A chain is the toxic agent, and the B chain binds to the target cell [1]. It is generally believed that anti-toxin neutralizing antibody (nAb) functions by blocking binding of the toxin to the cell, thinking that is enshrined in our teaching and in textbooks [2], [3], [4]. The implications of this belief include: 1. the B-chain would be the best target for vaccines and therapeutic Abs, and 2. that once toxin has entered cells, it is too late for nAb to function. These beliefs are based upon the elegant work of Pappenheimer [5], [6] with diphtheria toxin. Yet it was Pappenheimer himself who demonstrated that for the plant toxins abrin and ricin, Abs to both A chain and B chain neutralized and suggested that diphtheria toxin may be a unique case [7]. Since that time, the toxin-neutralizing ability of anti-A chain Abs has been clearly demonstrated [8], [9], [10], [11], [12], [13], [14], and for some toxins, including ricin and shiga toxins, anti-A chain Abs generally have greater in vitro neutralizing and in vivo protective activity than anti-B [13], [14]. The mechanism whereby Abs to A chain protect cells from toxins is now beginning to be elucidated [15].

We have previously produced a panel of anti-ricin monoclonal Abs (mAbs) to the A chain, B chain, and to determinants on both chains [14]. Although several mAbs neutralized ricin’s cytotoxicity and blocked its enzymatic activity in vitro, only one, RAC18, provided in vivo protection. Subsequent studies demonstrated that RAC18 afforded protection as late as 12–24 hr following a systemic or respiratory challenge with ricin [16], [17]. Here we use quantitative confocal microscopy, and other methods, to study the mechanisms of cytoprotection of RAC18 and other anti-A chain mAbs against the effects of ricin toxin. In the absence of Ab, ricin fully penetrates the target cells within 15–30 min. Intoxicated cells respond by blebbing and expelling the toxin. The results clearly demonstrate that rather than blocking the binding of ricin to the target cell, nAbs cause the accumulation of ricin at the cell surface, delay ricin internalization, and slow intracellular routing of the toxin to its target organelles. Ab continues to bind ricin intracellularly for hours. NAb can protect cells when administered even hours after exposure, when the toxin has fully penetrated the cell. These results demonstrate that nAb functions both extracellularly and intracellularly by altering internalization and trafficking of the toxin in the cell.

## Materials and Methods

### Reagents

Murine anti-ricin A chain mAbs RAC14, 17, 18, and 23 have been described elsewhere [14], as has the isotype control mAb 924 [18]. Hybridomas were grown in tissue culture in 10% low IgG fetal calf serum (FCS, Hyclone, Logan UT). A chimeric version of RAC18 was created by ligating genes encoding the murine RAC18 V-regions to human IgG1 (V_H_) or kappa (V_L_) C regions, and cloning each chain into pCDNA3.1 (Invitrogen). Plasmids were cotransfected into 293F cells (purchased from Invitrogen), and supernatants collected during the first 6 days in culture (C. Johnson and S. Pincus, unpublished). MAbs were purified from culture supernatant by protein G-sepharose chromatography (Sigma, St. Louis, MO). Ricin toxin was purchased from Vector Laboratories (Burlingame, CA). Alexa Fluors 488, 546, and 594 were obtained as N-hydroxysuccinimide (NHS) salts (Invitrogen Molecular Probes, Eugene, OR) and conjugated to ricin or RAC18 at a 5∶1 fluor to protein molecular ratio. Labeled protein was separated from unconjugated dye on Zeba 7KD cutoff desalting columns (Pierce, Rockford, IL). To avoid confusion, ricin is shown green in micrographs, regardless of which Alexa Fluor was conjugated. Biotin-LC-NHS (Pierce) and pHrodo-NHS (Invitrogen Molecular Probes) were conjugated to ricin in a similar fashion. Transferrin-Alexa 594, Brefeldin A BODIPY 558/568 (BFA-Bodipy), Lysotracker Blue DND-22 (LTB), Hoechst 33258, Annexin V-Alexa 488, and FM4-64FX were also purchased from Invitrogen Molecular Probes. Human cervical carcinoma (HeLa) and T-cell lymphoma (H9) cells have been serially passaged in RPMI 1640 medium, 10% FCS (Hyclone). The endoplasmic reticulum (ER) of HeLa cells was genetically marked by transfecting the cells with pDsRed2-ER (Clontech, Mountain View, CA), and selecting stable transformants in 400 µg/ml G418 (Sigma).

### Affinity Analyses

The affinity of mAbs RAC14, 17, 18, and 23 for ricin holotoxin was determined by surface plasmon resonance using Biacore 3000 (Piscataway, NJ). Abs were captured (50–150 response units) with anti-mouse IgG (Biacore) immobilized on CM5 sensor chips. Captured Ab was exposed to ricin in 0.1M lactose for 150 sec at 30 µl/min (association), followed by a dissociation phase of 1 hr, both at 37°. Chips were regenerated by removing the complexes with 0.01M glycine pH1.7. Ricin concentrations were optimized for each Ab and serial studies were performed at 2X dilutions of ricin (RAC18, 200 to 0.39 nM; RAC17, 1.6 µM to 3.13 nM; RAC23 and RAC14, 25.6 µM to 50 nM). Affinity of each Ab was measured in three separate experiments, and curves were fit to a 1∶1 Langmuir binding model with global Rmax.

### Toxin Neutralization Assay

HeLa or H9 cells, the latter a kind gift from Dr. M. Reitz, NCI [19] were plated into 96 well, flat bottom, tissue culture plates (Costar Corning, Lowell, MA) at 2X10^4^ cells per well in the presence of the Ab. Ricin was added 30 minutes later and the wells brought to a total volume of 200 µl. Two days later, 30 µl of MTS/PMS dye (Promega, Madison, WI) was added and A_490_ determined 3–5 hr later [14]. In experiments testing the effect of delayed administration of Ab, cells and ricin were added in a total volume of 150 µl, and then Ab added at the appropriate time in 50 µl.

### Light Microscopy

Wide-field images were obtained using a Leica DMRXA upright microscope, a 63X water-immersion objective, Sensicam QE CCD, and a Sutter Lambda 10-2 high-speed filter wheel equipped with the following filter sets: ex.D350/50x-em.D460/50m, ex.HQ480/40x-em.HQ535/30m, ex.HQ560/40x-em.HQ620/50m with respective polychroic mirrors. Time-lapse sequences were set, captured, and photobleach-corrected using Slidebook software (Intelligent Imaging Innovations, Denver, CO). All other images, including those used for quantitative analyses, were obtained with an inverted Zeiss LSM 510 microscope, a 63X 1.4 NA oil-immersion objective, and Zeiss LSM software. Heated (37°) stages and objectives were used on both microscopes. One day prior to imaging, 10^4^ cells were seeded into 35 mm culture dishes with 0.17 mm thickness glass bottom (MatTek, Ashland, MA). Cells were cultured at 37° in RPMI 1640 with no phenol red, 10% FCS. The following day, cells were placed into RPMI 1640, 1% bovine serum albumin (BSA), and 10 mM Hepes, and transferred to the microscope stage for imaging. Hoechst dye (2 µg/ml) and BFA-bodipy (250 ng/ml) were added prior to ricin, LTB (125 nM), FM4-64 (5 µg/ml), and transferrin-Alexa 594 (20 µg/ml) at the same time as ricin +/− Ab. Fluorophore-conjugated ricin was added to a final concentration of 3.6 µg/ml. BFA-bodipy was tested to demonstrate that at the concentration used there was minimal effect of the BFA on the toxicity of ricin. BFA-bodipy was used in only one set of experiments to demonstrate rapid accumulation of ricin in Golgi and ER. It was not used in any quantitative analyses. Micrographs shown in this manuscript have been enhanced by adjusting brightness and contrast so that differences are more clearly grasped by the human eye. Identical brightness/contrast settings are used for images compared to one another. Quantitative analyses have been performed on raw, uncompensated data. No deconvolution algorithms have been applied to images.

**Figure 1:**
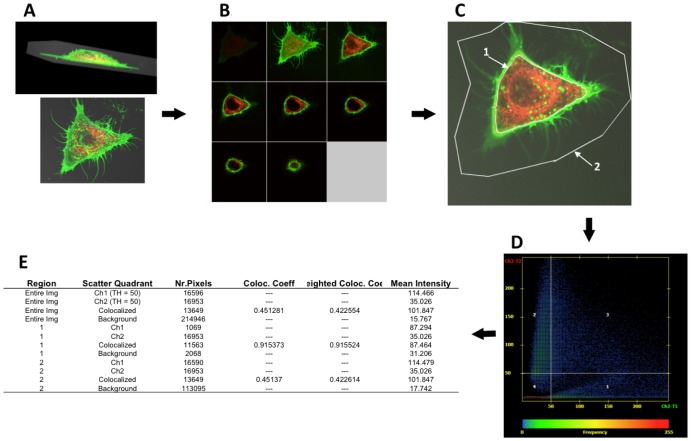
Demonstration of methods for quantifying confocal imaging data. A. Cells were imaged as 1 µm vertical stacks at different times following the administration of ricin (always green in all figures). The cells were genetically modified to express DsRed in the endoplasmic reticulum. B. Each plane was viewed separately in this display. C. The third vertical image (upper right in panel B) was chosen for analysis. Two ROI’s were drawn: ROI 1. contains the intracellular ricin, ROI 2. defines the total amount of ricin associated with the cell. D. and E. are graphical and mathematical representations of the visual data.

Our general scheme for quantitative analysis of confocal micrographs is shown in figure 1. In each experiment, a series of different cells were studied with time after the addition of ricin, generally 25–30 observations during the 60 min period. Attempts to obtain multiple images from the same cell induced a much higher rate of blebbing than observed in cells in ricin the same amount of time, but only imaged once. We assume this “hyperblebbing” was the result of combined phototoxicity and ricin effect, and thus imaged each cell only once to minimize the combined toxicities. For each curve, a minimum of three independent experiments were performed and the results pooled into one data set for statistical analysis. Laser, microscope, and PMT settings were established in preliminary experiments and remained unchanged through the entire series of experiments presented in each graph. Data were collected at 8 bit depth, and although machine settings aimed to maximize the full dynamic range, care was taken to avoid oversaturation (ie pixels reading in channel 255). For each cell, a Z-stack with 1 µm steps was imaged (figure 1A and B). Pinhole settings were such that an optical slice was <1 µm. A single plane approximately 1/3 of the height from the bottom to the top of the cell stack was chosen for analysis (top right section in figure1B). Using the AxioVision (Zeiss) analysis module, two regions of interest (ROI) were drawn on each section by observers blinded to experimental conditions (figure 1C). The first ROI contains the internal contents of the cell and is defined by the dense deposition of ricin (green) at the cell surface. Any ricin protruding into the cell, yet in contact with ricin on the surface is considered external. The second ROI was drawn to contain all ricin associated with the cell, inside and out. Figure 1D shows a dot plot obtained by graphing green vs red image intensity for each pixel in the section. To eliminate noise from low level intensity readings of background pixels, pixels with an intensity of <50 (out of 255) were excluded from data analysis (1D). For each ROI, the number of pixels, the mean intensity, and degree of colocalization were calculated using AxioVision (1E). From these data we calculated the proportion of cell-associated ricin that has been internalized and the degree of ricin colocalization with the second marker. Percent internalization was determined as the fraction of total ricin intensity inside the cell (ROI 1) divided by the total ricin intensity inside and outside of the cell (ROI 2), where total ricin intensity is measured as the sum of the above-threshold pixel values within a given ROI. We calculated the degree of colocalization of ricin as the Pearson correlation coefficient between ricin-channel pixel intensity and marker-channel pixel intensity, calculated over all image pixels exceeding the threshold value for both ricin and the colocalizing marker.

The percent internalization and the correlation coefficient were plotted against time, fitted to curves, and analyzed statistically using Prism (GraphPad Software, La Jolla, CA). The data were fitted with a nonlinear regression using the formula:
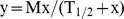
where M is the maximal value and T_1/2_ is the time to reach the half maximal value. This equation was chosen because it yields the closest fit (R^2^) of the simple models. Although it is the Michaelis-Menten equation, we do not mean to imply that the observed events are the result of a single enzymatic step. Statistical significance of differences between curves was determined using the *F* test.

Fluorescence recovery after photobleaching was performed on a Zeiss 710 microscope, using Alexa 488-labeled ricin. Three images were obtained prior to photobleaching. A region of 14×15 pixels (0.21 micron/pixel) was bleached with a 488 laser line at maximum power with a 1.58 microsecond pixel dwell time for 10 iterations. Images were obtained following the bleach cycle and every second thereafter for 2.5–3 min. Laser intensity during image acquisition was minimized to avoid continued bleaching with the repetitive analyses. Data was analyzed as described above, using the same equation to fit a nonlinear regression, except in this case M is replaced by the maximal value for fluorescence recovery (R_max_).

### Electron Microscopy

Aclar film (Ted Pella, Redding, CA) was cut to 0.7 cm circles and placed into 24 well TC plates (Costar). HeLa cells were plated at 4×10^4^ cells/well and grown 48 hr in RPMI 1640 and 10% fetal calf serum. The wells were then rinsed with serum free RPMI, and cells resuspended in RPMI containing 1% BSA. Biotin-conjugated ricin or an irrelevant biotin-conjugated protein were added at 6 µg/ml, the cells incubated for the indicated time at 37°, washed for 10 min ×3 in 0.1M sodium cacodylate buffer, and fixed in 2% freshly opened glutaraldehyde in 0.1M sodium cacodylate (both from EM Sciences) for 1 hr. The cells were then washed again in cacodylate buffer (10 min ×3), and then PBS. Wells were incubated for 1hr with ABC Elite (Vector Labs), washed in PBS, and stained with diaminobenzidine (Sigma) 0.5 mg/ml plus 0.03% H_2_O_2_ for 10 min, before washing in PBS, and then distilled water. Cells were post-fixed for 20 min in 1% osmium tetroxide, washed in water, dehydrated with 50%,70%,80%,95%, and 100% ethanol, then run thru a series of ethanol-plastic solutions containing increasing concentrations of plastic; and placed in pure plastic consisting of 25 parts eponate 12 resin (Ted Pella), 20 parts araldite 502 resin (Ted Pella), 60 parts dodecenyl succinic anhydride, (EM Sciences, Hatfield PA), and 2.1 parts DMP-30 (EM Sciences) for 1.5 to 2 hr, and polymerized for 24–48 hr at 60°C. Ultrathin sections were cut and collected on copper mesh grids. The sections were imaged at 80 kV on a Hitachi H-7500 transmission electron microscope.

### Flow Cytometry

To quantify the effects of Ab on ricin binding to cells, H9 cells were mixed with Ab (10 µg/ml), then ricin-Alexa-488 (3.6 µg/ml) was added. Cells were incubated for various times either at 37° in RPMI 1640+1% BSA, or at 4° in PBS +1% BSA+sodium azide (0.1%). Following the incubation, cells were washed ×2 in PBS, and fixed in 4% paraformaldehyde (Sigma) in PBS pH7.4. For analysis of cell surface markers, cells were incubated with primary and then secondary Abs in PBS/BSA/Azide. For Annexin V/propidium iodide analyses, viable cells were stained and analyzed. Ten thousand cells were analyzed in the Fl1 (FITC) and/or Fl2 (PE) channel of an LSR II flow cytometer (Becton Dickinson, San Jose, CA) and FlowJo software (Treestar, Ashland, OR). Displayed histograms are based upon cells gated for SSC and FSC.

## Results

### Localization of Ricin within Cells

**Figure 2:**
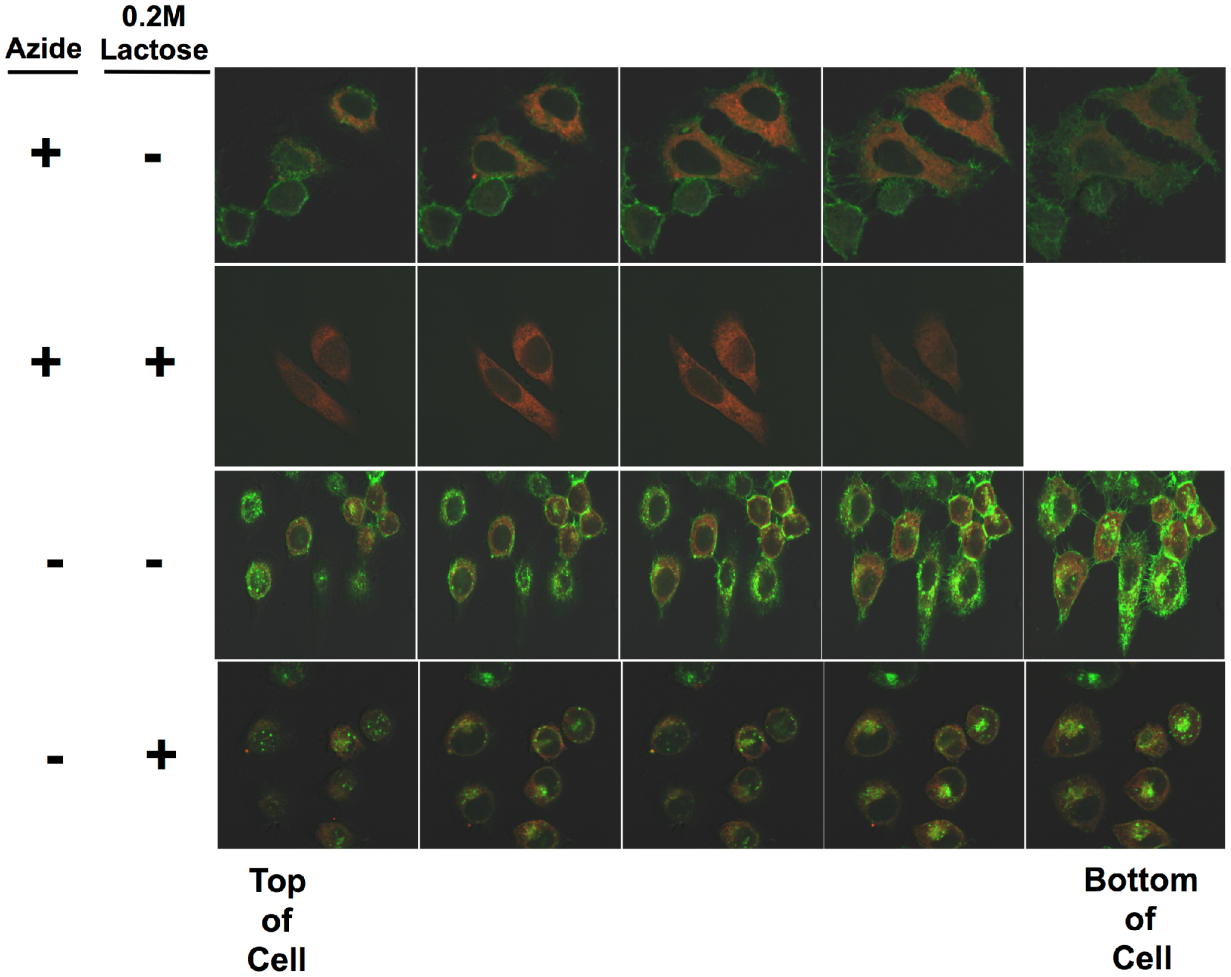
Internalization of fluorescent ricin. HeLa cells stably transfected with pDsRed-ER were plated on glass coverslips and incubated in tissue culture. One day later, half of the slides were washed with cold PBS, 1% BSA, 0.01% sodium azide and incubated in the same, on ice. The remaining slides were left under physiological conditions. Thirty min later, Alexa 488-conjugated ricin was added to each set of slides and the slides were incubated an additional 45 min under the same conditions as previously. Following this, cells were washed 3X with ice cold PBS/BSA/Azide. Cells were then incubated in either PBS or PBS/0.2M lactose for 5 min with orbital shaking. The solution was removed, fresh PBS or PBS/lactose added, and the process repeated three times. The cells were fixed in 2% paraformaldehyde. Cells were viewed with a 62X oil-immersion objective. Each panel shows a Z stack, each plane separated by 0.8 µm. The plane closest to the slide (bottom of the cell) is to the right. Ricin is green, ER red. There is no nuclear stain.

**Figure 3:**
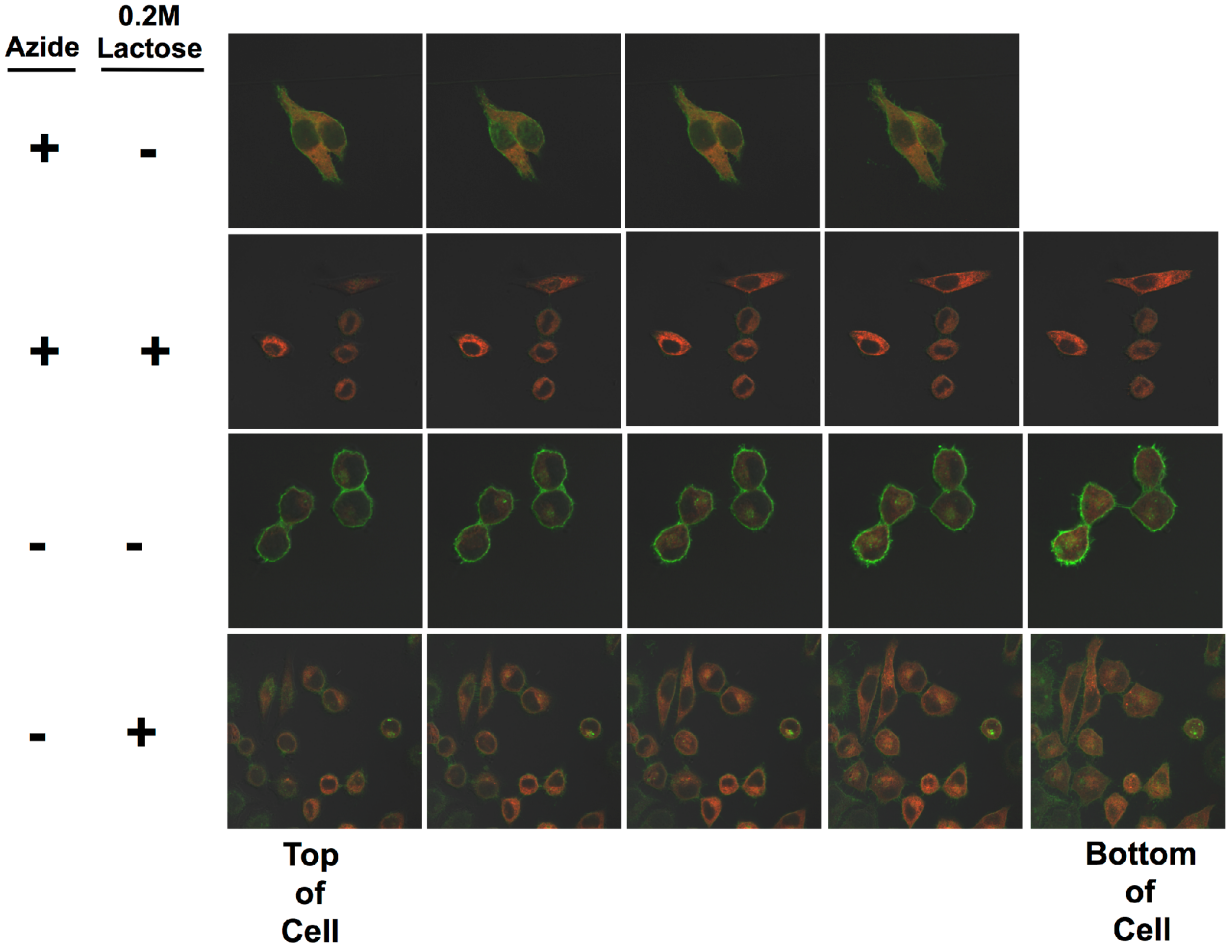
Internalization of ricin/mAb immune complexes. Experimental conditions are as described in figure 2, except in this case the cells were first incubated with unlabeled ricin, washed, and then with Alexa 488-conjugated RAC18, either in the cold with azide, or at 37^0^ in tissue culture medium.

In the studies presented here we utilize quantitative confocal microscopy to study the effects of neutralizing Ab on the internalization and intracellular routing of ricin toxin. In figure 1, we show our basic approach to quantifying ricin internalization. In figure 2 we demonstrate the internalization of ricin. HeLa cells expressing the ER-localizing DsRed protein were incubated with Alexa 488-conjugated ricin under physiologic conditions, or to block the entry of ricin into the cell in the cold with sodium azide. The cells were then washed, and either treated with 0.2M lactose to remove cell-surface ricin [20], [21], or not. Serial vertical sections are shown in figure 2. In the presence of azide, top two panels, ricin only binds to the cell’s surface, and can be completely removed with 0.2M lactose. When ricin is incubated with cells at 37^0^ in tissue culture medium, considerably more ricin is associated with each cell, and only a portion of the cell-associated ricin is removed by exposure to lactose, indicating that much of the cell-associated ricin is not surface accessible. A similar experiment is shown in figure 3, but in this case, it is the internalization of fluorescent RAC18 mAb that is shown. Cells were first incubated with unlabeled ricin for 30 min, washed, and then with labeled RAC18, under the indicated conditions. No binding of RAC18 was observed in the absence of ricin (not shown). As with ricin, in the presence of azide, all Ab is removed with lactose, and thus is cell surface accessible, whereas under physiologic conditions Ab is internalized and only a portion can be removed by washing cells with lactose.

**Figure 4:**
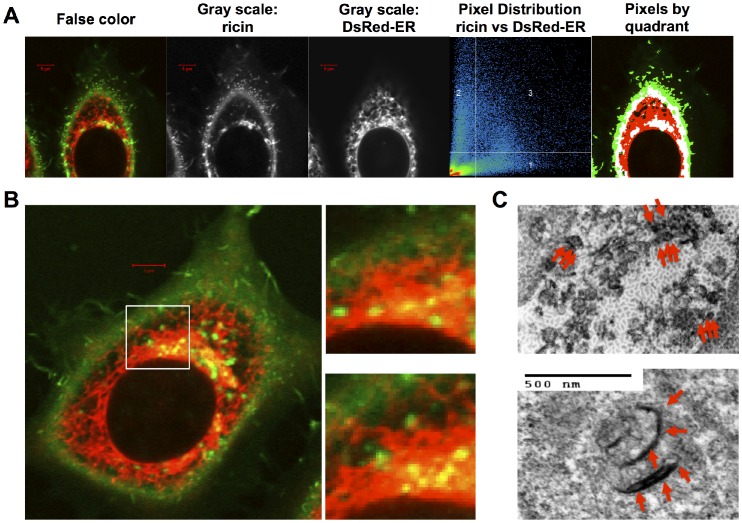
Localization of ricin in ER. In panel A five different views of the same image are shown, demonstrating the colocalization of ricin (green) and Ds-Red ER. The left panel shows a false color image of both markers, followed by two gray scale images showing the ricin and ER separately. The fluorescent intensity of each pixel is mapped in a 2-D dot-plot showing red on the vertical axis, and green on the horizontal axis. The right hand image maps the micrograph by quadrant of the pixel in the dot plot: pixels in quadrant one are green (ricin only), quadrant 2 are red (ER only), colocalized pixels are white (quadrant 3), pixels that fall below the threshold for both fluors are black. Panel B shows a false color confocal image, with a 10 µm×10 µm region marked. To the right are two vertical planes from that region, vertically separated by 0.7 µm. Colocalized pixels are yellow-orange. In panel C we show TEMs demonstrating HRP-ricin (red arrows) localized in the ER and Golgi.

Biochemical and electron microscopy studies by Sandvig, Nicolson, and others, showed that ricin enters endocytic vesicles and vesiculo-tubular elements in 5–30 minutes, and can be observed in the Golgi within 15 minutes. By 30–60 minutes ricin localized to the perinuclear region rich in Golgi and ER. These earlier studies disagreed as to whether ricin could be detected free in the cytoplasm [21], [22], [23], [24], [25], [26]. Having shown that we can detect intracellular ricin, we asked if ricin in specific organelles could be setected by confocal microscopy. Measuring proximity of two molecules by fluorescence microscopy may be performed in several ways. The most direct is colocalization. But it is limited by pixel size, depth of vertical focus (optical slice), and the physical limitations of optical resolution. Colocalization is operationally defined as two fluors exceeding a given threshold intensity within the same pixel. Because vertically separate labels can colocalize if the depth of focus is too great, colocalization is best studied by confocal microscopy. Colocalization of Alexa-488-ricin with DsRed marked endoplasmic reticulum is shown in figure 4. In figure 4A five different views of the same image are shown, demonstrating the colocalization of ricin (green) and Ds-Red ER. We show from the left: false color image of both markers, two gray scale images showing the ricin and ER separately, a 2-D dot-plot of each pixel with red intensity on the vertical axis and green on the horizontal axis. The right hand image maps the micrograph by quadrant of the pixel in the dot plot. Colocalized pixels are best shown in the right hand figure. Figure 4B shows a false color image of a cell. Two serial vertical images of the region marked by the inset are enlarged. The z-axis separation of the images was 0.7 µm. These show distinct regions of red, green, and yellow, the latter indicating colocalization. The patterns of colocalization are different in the adjacent slices, indicating that this is true colocalization, and not visual stacking of separated colors into one vertical slice. Figure 4C shows immunoperoxidase stained ricin in ER and Golgi by TEM.

### Ricin is Rapidly Internalized and Distributed through the Cell; it is Primarily Eliminated via Blebbing, with Internalization and Expulsion Reaching a Steady State within 1 hr

**Figure 5:**
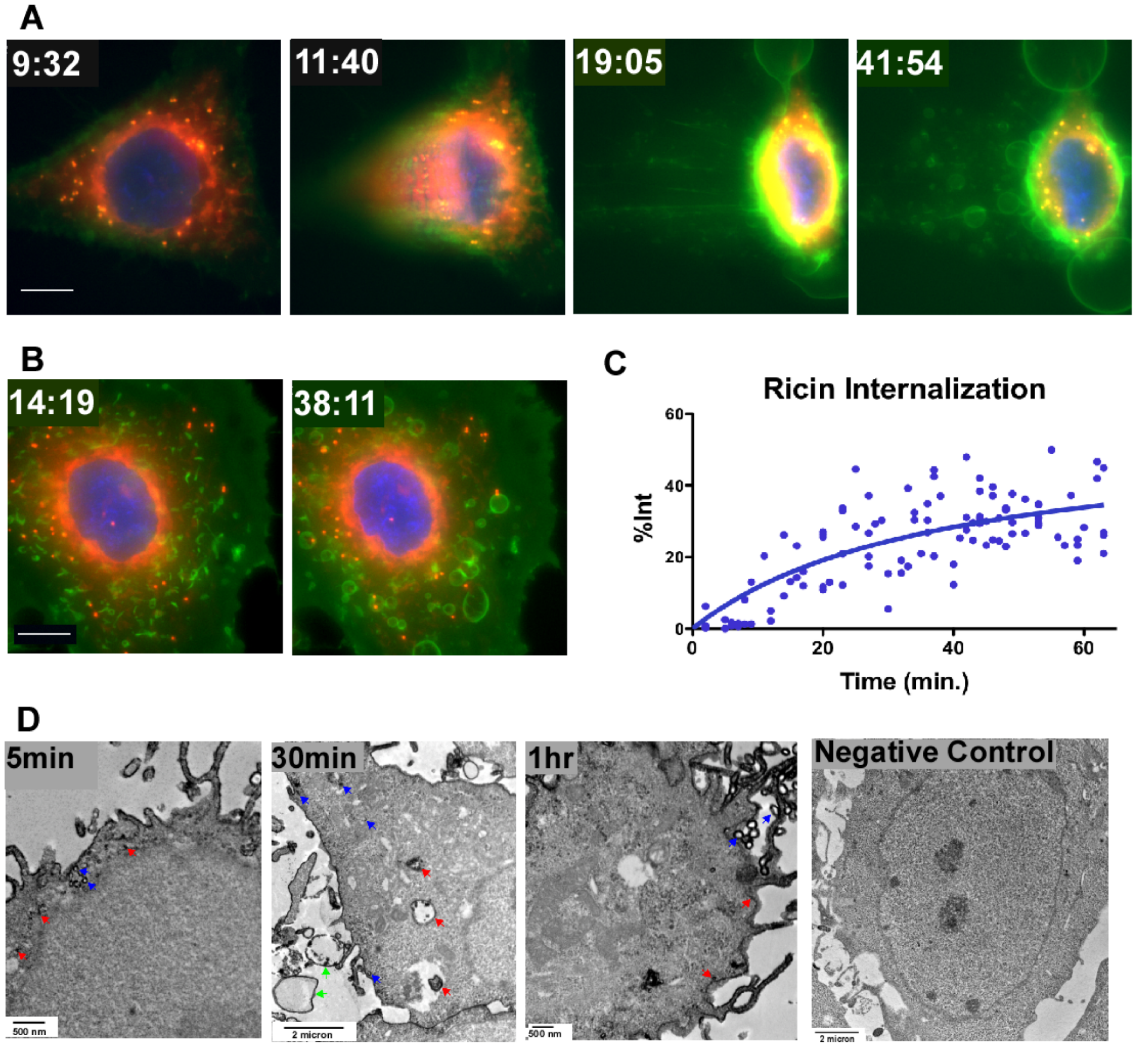
Ricin enters cells and attains wide intracellular distribution within one hr. A. and B. Two different live cells were repeatedly imaged with a wide field water immersion objective. Nuclei were stained with Hoechst dye (blue), ER and Golgi are stained with Bodipy-brefeldin A (red), and ricin (green) was added at 4 min. The white bar indicates 10 µm. The full time series are shown in videos S1 (A) and S2 (B). Time is indicated in min and sec. This degree of blebbing has been confirmed in >10 other time series micrographs. C. Percent of cell-associated ricin that has entered the cell was determined for 105 different cells in three separate time course experiments. Dots represent individual determinations, the curve was obtained using the model described in Methods. D. TEMs show ricin as electron-dense material and reveal ricin in the cell within 5 min and ricin coating vesicles and/or blebs by 30 min. Arrows indicate examples of cell-associated ricin accumulation. Images are representative of >100 images collected in two different experiments, each with up to 4 replicate samples. Figure 6 demonstrates that cell death does not occur until 24 hr.

**Figure 6:**
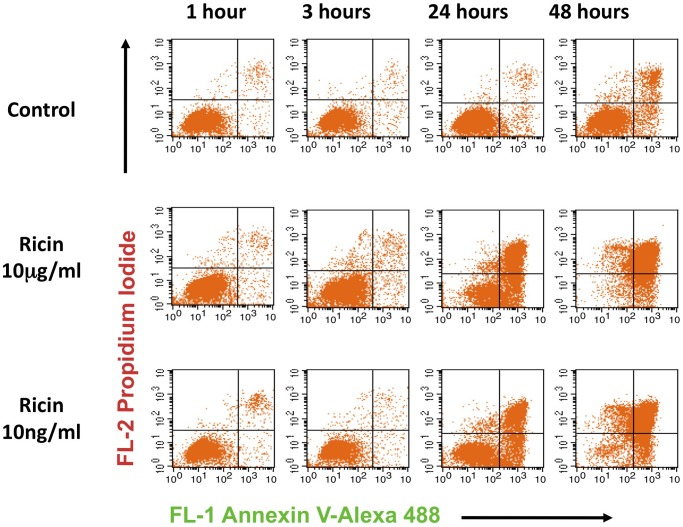
Development of apoptosis and cell death following administration of ricin. Live cells were incubated with ricin and at the indicated time points were stained with propidium iodide and Annexin V-Alexa 488, and analyzed by flow cytometry 15–30 min later. Propidium iodide is excluded from viable cells. Annexin-V binding is a measure of apoptosis. Thus cells can be considered healthy (lower left quadrant), dead by non-apoptotic mechanisms (ULQ), undergoing early apoptosis (LRQ) or dead by apoptosis (URQ). Significant increases in apoptotic or dying cells does not occur until 24 hr. These experiments have been repeated >3 times with similar results.

We first studied the binding, internalization, and intracellular distribution of ricin in the absence of Ab (figure 5). Figures 5A and 5B show images of single cells that were stained with Hoechst (nucleus, blue) and Bodipy-BFA (Golgi and ER, red). Ricin (green) was added at 4 min. The full sequence of events, demonstrating the intensity of blebbing, is shown in videos S1 (of the cell in fig 5A) and S2 (5B). In 5A, the binding of ricin to the cell is evident within 5 min. At 7 min following administration, ricin has entered the cell, and within 15 min has spread throughout. The adherent cell in figure 5A has partially detached from the tissue culture plate and the side view shows blebs forming and moving away from the body of the cell. Figure 5B shows the formation and expansion of ricin coated blebs. As noted in the Materials and Methods, repeated exposure of these cells to light may result in an increased degree of blebbing. To avoid this, in the quantitative analyses shown below (excepting the FRAP studies), individual cells were exposed to light only one time. We have quantified the internalization of ricin versus time (figure 5C). The data show the proportion of cell-associated ricin that has entered the cell. Each point represents an individual measurement, each performed on a different cell. Within one hr, the cells approach a steady state where the amount of ricin internalized is roughly equivalent to that which has been expelled from the cell, results that are consistent with the previously published work of Sandvig and others [27], [28]. Figure 5D shows events at an ultrastructural level, where ricin is identified as electron-dense material. At 5 min ricin can be seen entering the cell in small vesicles (blue arrows) and is localized in cytoplasmic regions near the cell membrane (red). At 30 min ricin still lines the cell surface, and is observed on internal membranes (blue). Within the cell is a series of large vacuole-like structures either coated with or containing ricin (red). We cannot determine if this is ricin entering or exiting the cell. Large blebs coated with, and in some cases containing, ricin are observed outside of the cell (green). At one hr ricin is well distributed throughout the cytoplasm, multiple blebs are seen along the cell surface (blue), but ricin continues to enter the cell (red). It seems likely that the blebbing results in the expulsion of ricin from the cell. Data supporting this conclusion include: ricin-containing debris found within the blebs (figure 5D), the presence of ricin-coated blebs and cellular material adjacent to, but clearly outside, the cell, and the steady state obtained between income and outgoing ricin (figure 5C). Despite this rapid internalization and dissemination of ricin, and the apparent disruption of cellular functions by blebbing, apoptosis does not occur until 24 hr (figure 6).

### In the Presence of Neutralizing Ab, Ricin Accumulates at the Cell Surface

**Figure 7:**
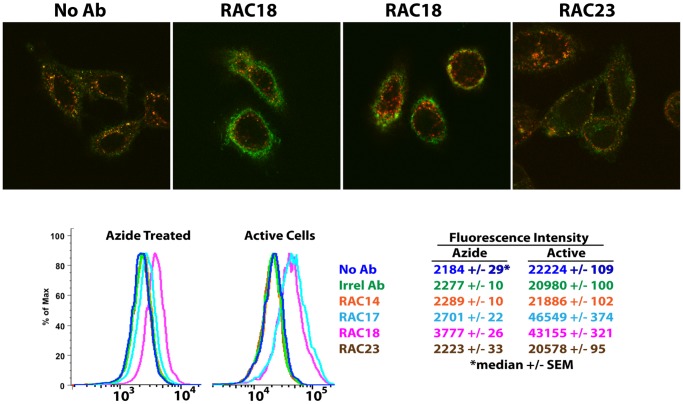
Ricin accumulates at the cell surface in the presence of neutralizing Ab. Top. Live cells were incubated with labeled transferrin (red) and ricin (green) for 10 min in the presence of the indicated antibodies, or no antibody, and then fixed. Cells were imaged via confocal microscopy, and a central slice from a z stack is shown. In the absence of Ab (left), ricin and transferrin enter the cells by similar processes and colocalize. But in the presence of the nAb RAC18 (center), ricin is retained at the cell surface, while transferrin still enters. Non-neutralizing Ab RAC23 (right) has no effect on ricin internalization. Full z stacks of each of these photos are shown in videos S3, S4, S5, S6. These experiments have been replicated in three individual experiments, with >100 cells visualized. Bottom. The increase of cell surface ricin is confirmed, and quantified, by flow cytometry. Cells were incubated with the indicated Ab and ricin for 60 min either in the cold with sodium azide, or at 37° in tissue culture medium. The former studies passive binding at the cell surface, the latter also allows for internalization. 10,000 cells were analyzed. In either case RAC 18 results in approximately two-fold increase in cell-associated ricin.

We have previously evaluated the neutralizing abilities of a large panel of anti-ricin A chain Abs [14]. Ab RAC18 blocks ricin’s ribosome N-glycosidase activity, neutralizes ricin’s cytotoxicity and is highly protective in vivo, whereas RAC23 binds well and blocks ricin’s enzyme function, but neither neutralizes in vitro nor protects in vivo. To study the effect of nAb on the internalization and intracellular localization of ricin, we exposed live cells to labeled ricin (green) and labeled transferrin (red) in the presence of RAC18, RAC23, or no Ab. Ricin and transferrin both bind to surface proteins (transferrin uses a specific receptor), are internalized with similar kinetics, and enter cells via endosomes. HeLa cells were incubated for 10 min at 37°, fixed, and examined by confocal microscopy (figure 7 top, videos S3-S6). Neither of the Abs alters the internalization of transferrin. In the presence of no Ab or RAC23, ricin and transferrin largely colocalize. But in the presence of the nAb RAC18, ricin remains at the cell surface and has limited entry into the cell, while the internalization of transferrin is unaffected. To confirm that nAb increases the amount of cell-associated ricin, we have performed flow cytometry, either in the presence of 0.01% sodium azide at 4°, so that only binding of ricin to the cell surface was measured, or for 1 hr at 37° in standard tissue culture medium, to allow for both surface binding and internalization (figure 7 bottom). In addition to RAC18 and RAC23, other Abs were used: RAC 17 (neutralizing, but no in vivo protection), RAC 14 (non-neutralizing), and an isotype control Ab. The results show that RAC18 increases both surface and total cell-associated ricin, RAC17 also increases total cell associated ricin, and none of the other Abs has any effect. Contrary to what would be expected if neutralizing Ab blocks binding of toxin to the cell, there is an increase in ricin accumulation, predominantly at the cell surface. NAb may cause this accumulation by forming immune complexes at the cell surface, or ricin may accumulate because it’s entry into the cell is hindered.

### Neutralizing Ab Delays Internalization and Cellular Distribution of Ricin

**Figure 8:**
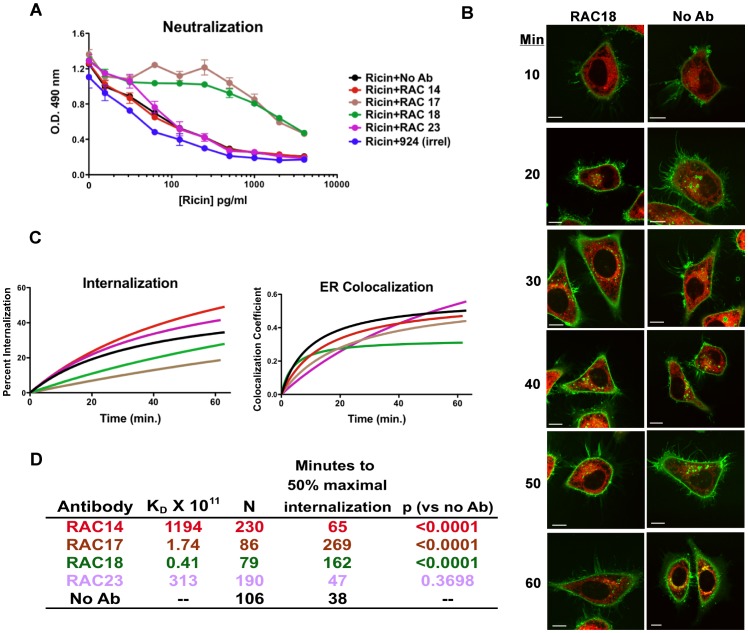
Neutralizing Ab delays internalization of ricin. A. Neutralization of ricin cytotoxicity by mAbs is confirmed using MTS assays. H9 cells were premixed with Ab at 10 µg/ml, and then ricin was added to the indicated concentration. MTS dye reduction was measured 48 hr later. This is representative of >5 Ab neutralization assays, error bars indicate SEM. B. HeLa-DsRed cells were incubated with labeled ricin (green) for the indicated time in the presence or absence of RAC18 mAb and images obtained by confocal microscopy. The white bar indicates 10 µm. C. Quantitative analyses of internalization and colocalization of ricin. Data are collected from confocal images, including those shown in panel B. At least three distinct experiments were performed and the number of individual data points collected is indicated in the table in panel D. Colors of curves conform to colored lettering in the table. D. Affinity of Abs, and data obtained from internalization curves shown in panel C. Affinity was determined by Biacore analyses of mAbs captured via Fc and tested against soluble ricin. N is the number of cells imaged to obtain the data for analysis, and the p value (by F test) compares the internalization curve of each Ab to that of no Ab.

For ricin to kill a cell, it must traffic through a well-defined pathway to its site of toxic action. To determine what effect nAb has on this trafficking, we examined the relationship between neutralization, toxin internalization, and colocalization using the panel of anti-ricin A chain Abs (figure 8). We first confirmed the in vitro neutralization activity in HeLa cells of the preparations of Abs used in the subsequent studies (figure 8A). As expected RAC17 and RAC18 effectively neutralized ricin, while RAC14 and RAC23 did not. We then studied the entry and localization of fluorescent ricin in HeLa cells with the ER marked with DsRed fluorescent protein (figures 8B–D). Live cells, maintained on a heated microscope stage, were incubated in the presence of the different Abs and fluorescent ricin. Different cells were imaged as z-stacks over time, up to 1 hr following the addition of ricin. Figure 8B shows confocal micrographs taken at different times following the addition of ricin, in the presence or absence of neutralizing Ab. Membrane-associated ricin was present from the earliest time points, and with time increasing amounts of ricin entered the cells and colocalized with the ER. There appears to be less internalization and colocalization in the presence of nAb. To quantify this, we measured the rates of internalization and ER colocalization (figure 8C, showing the curves, and 8D, quantitative descriptors). Ab binding constants to ricin holotoxin were determined by surface plasmon resonance (figure 8D). RAC17 and RAC18, the two neutralizing Abs, had the greatest affinity. Panel 8D also shows T_1/2_ for ricin internalization and the statistical significance of the difference between the ricin internalization curves measured in the presence or absence of Ab. The results include all data obtained in at least three distinct experiments, in the presence and absence of Ab. The total number of data points is shown in figure 8D. The results clearly demonstrate a significant delay in the internalization of ricin in the presence of the high-affinity neutralizing Abs (RAC17 and RAC18, curves in C below no Ab), but not non-neutralizing Abs (RAC14 and RAC23, with RAC14 showing significantly more internalization with time than no Ab). The results showing the kinetics of ER colocalization are more difficult to interpret. RAC18 has the highest initial rate of colocalization, but attains the lowest overall degree of colocalization (figure 8C), whereas RAC23 is just the reverse, lowest rate of internalization, but attaining the highest degree of colocalization. RAC14, RAC17, and no Ab lie between these extremes. One possible explanation for this observation is that binding by RAC18 directs more of the ricin to degradative pathways.

**Figure 9:**
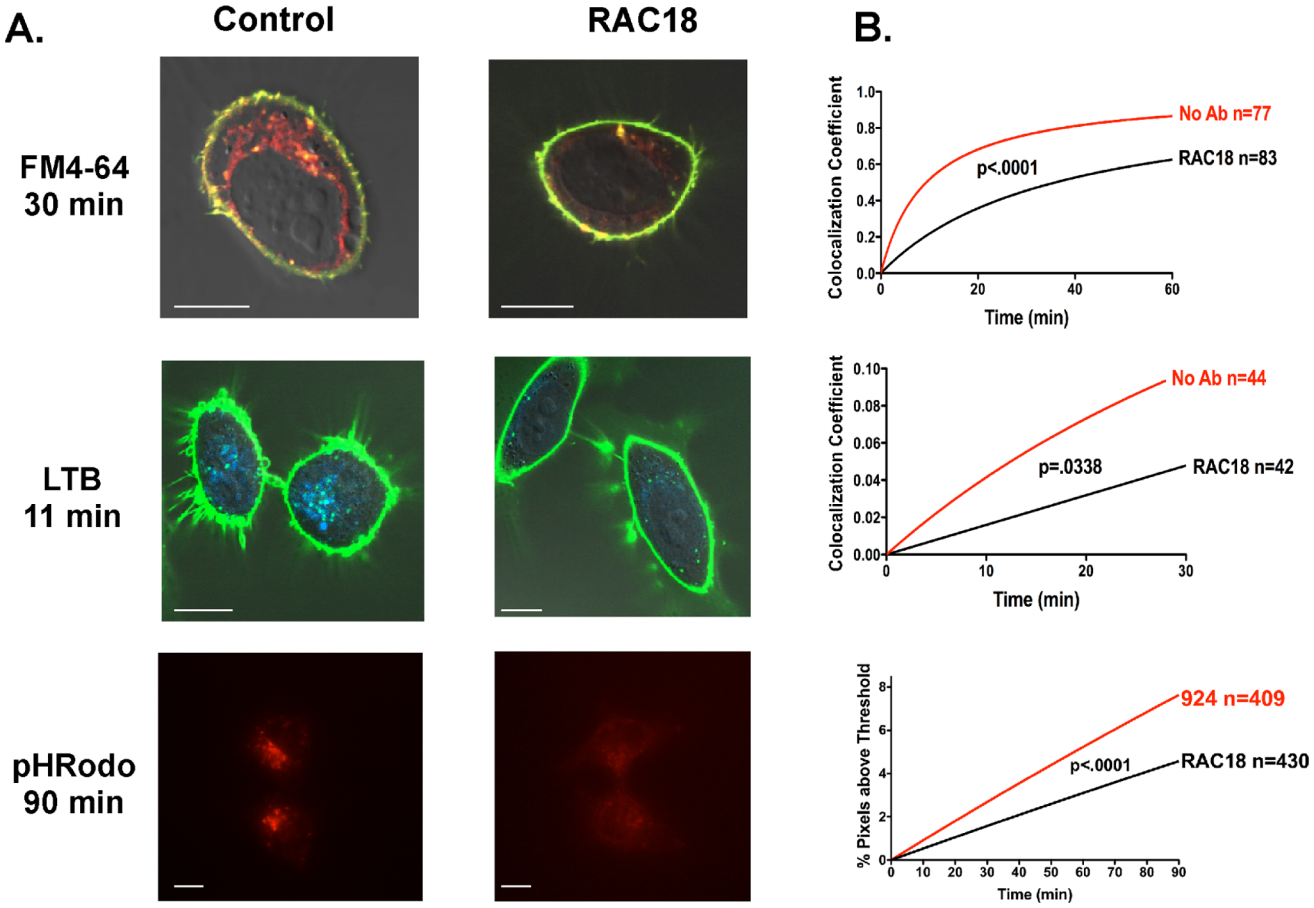
Effect of nAb on intracellular localization of ricin. Panel A shows original micrographs, B shows the curves extracted from such micrographs of live HeLa cells, imaged over time. The white bars on graphs indicate 10 µm. Indicated on the graph are the number of individual cells analyzed (in 3 or 4 distinct experiments) to obtain each curve, and the results of statistical comparisons of the curves. FM4-64 is a lipophilic dye, initially binding to the cell surface membrane, but then internalizing and become associated with intracellular lipid membrane structures. LTB is an acidophilic dye that initially accumulates in liposomes, but eventually also stains nuclei. For both FM4-64 and LTB, the proportion of ricin colocalizing with the dye is graphed. pHrodo was conjugated to ricin (and is shown in red). At acidic pH, the fluorescence of pHrodo is markedly enhanced. Thus for pHrodo, a threshold intensity was established at 100 (out of 256). The number of pixels exceeding that intensity were counted and plotted as percent positive within an ROI encompassing the entire cell.

To further examine the effect of nAb on ricin’s intracellular localization, colocalization analyses (figure 9) were performed with two dyes: 1. the lipidophilic dye FM4-64, which initially binds to the cell’s plasma membrane, but soon translocates to internal membranes as well, and 2. LysoTracker Blue (LTB) an acidophilic dye that initially binds to lysosomes, but begins to accumulate in the nuclei within 20–30 min. Individual photomicrographs, taken at the times indicated, are shown in panel A and colocalization curves in B. For both dyes, the presence of nAb results in less colocalization. Both ricin and FM4-64 enter the cell together in the absence of Ab, but in its presence ricin is retained at the cell surface, while FM4-64 enters. To measure ricin’s localization in acidic vacuoles, ricin was labeled with the pH sensitive dye, pHrodo. Fluorescence intensity increases with falling pH, and thus bright fluorescence is associated with localization of the ricin in acidic compartments, most notably the phagolysosome. Using a gated threshold to measure acidification, we again see that neutralizing Ab delays the entry of ricin into acidic vacuoles. Together, the data in figures 8 and 9 indicate that nAb delays the entry of ricin into the various pathways it must traverse en route to its site of toxic action, the ribosome.

**Figure 10:**
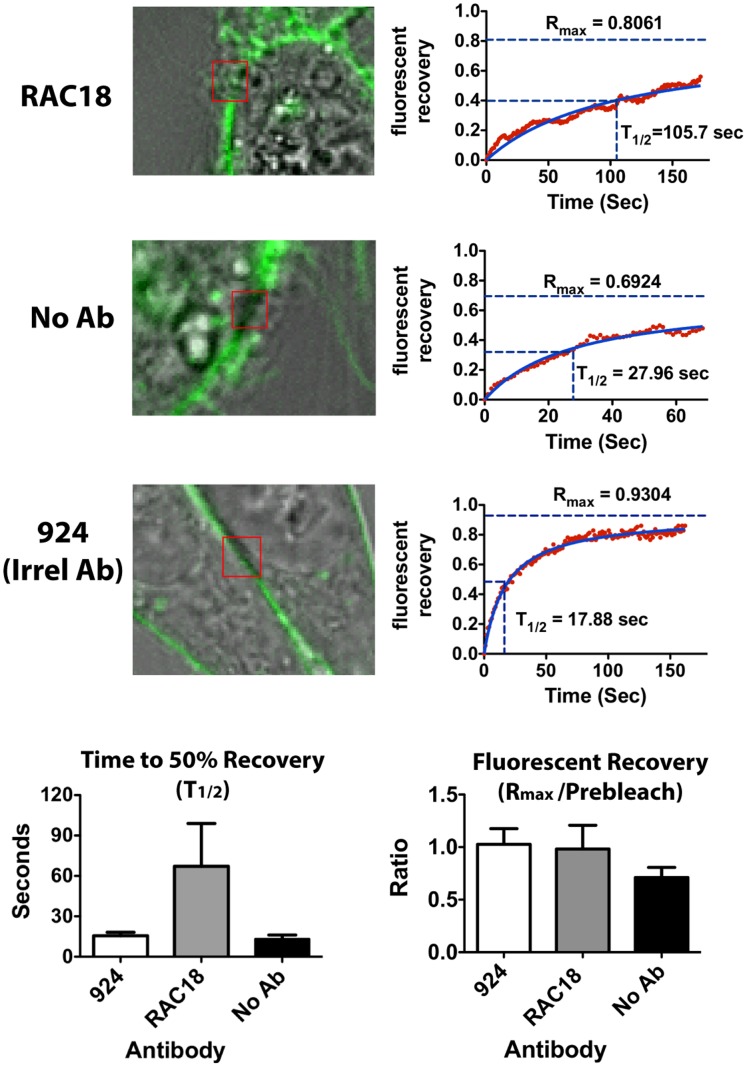
Use of fluorescence recovery after photobleaching to study effect of Ab on mobility of cell membrane ricin. Live cells were incubated with ricin-alexa 488. A region of the cell membrane was photobleached. Fluorescence in that region was measured prior to bleaching, and every second during the recovery phase. Top left: Photomicrographs of cells immediately following photobleach. The red square (2.94×3.15 µm) indicates the region that was bleached and measured. Top right: The fluorescent recovery is plotted versus time. Each red dot is an individual measurement. Videos S7, S8, and S9 show micrographs of the entire time series for each of the cells shown. The blue curves, R_max_, and T_1/2_ are derived from the same model used to calculate percent internalization and correlation coefficient. These curves were obtained from serial images of the cells shown to their left. Bottom. Statistical analyses of samples run in the presence of nAb, control Ab, or no Ab. Results are mean and SEM of six or seven independent analyses for each condition. Differences were not statistically significant.

One manner in which nAb could slow internalization might be by hindering the movement of the ricin/cell-surface protein complex through the cell membrane to sites of internalization. Ab may bind bivalently to two ricin molecules attached to different cell-surface structures, markedly increasing the size of the complex, and increasing its resistance to movement in the membrane. To measure Ab effects on ricin membrane mobility, we measured fluorescence recovery after photobleaching (FRAP). A membrane region of cells incubated with fluorescent ricin was photobleached to approximately 30% of the initial fluorescence, and the fluorescent intensity within the region was measured with time post-bleach, as unbleached ricin moved into the region (figure 10). Photomicrographs of cells, in the presence or absence of nAb, are shown post bleaching in the top left of figure 10. To the right, graphs show the mean fluorescent intensity of that cell’s bleached region plotted against time. Videos S7, S8, and S9 show time series micrographs. At the bottom, we combine the results of 6 or 7 replicate FRAP experiments. The T_1/2_ is a function of lateral mobility, whereas the presence of a large immobile fraction (represented as the ratio of R_max_ to prebleach fluorescent intensities) would indicate compartmentalization [29], [30]. The T_1/2_ value in the presence of Ab is 3X that in its absence, although the results are not statistically significant. This suggests that the formation of macromolecular complexes at the cell surface, containing Ab, ricin, and ricin-ligands, likely results in impaired mobility of ricin (and its ligands) in the cell membrane, and that this might contribute to the observed delay in internalization. There is no evidence of compartmentalization of cell-surface ricin.

### Ab and Ricin Remain Colocalized Inside the Cell

**Figure 11:**
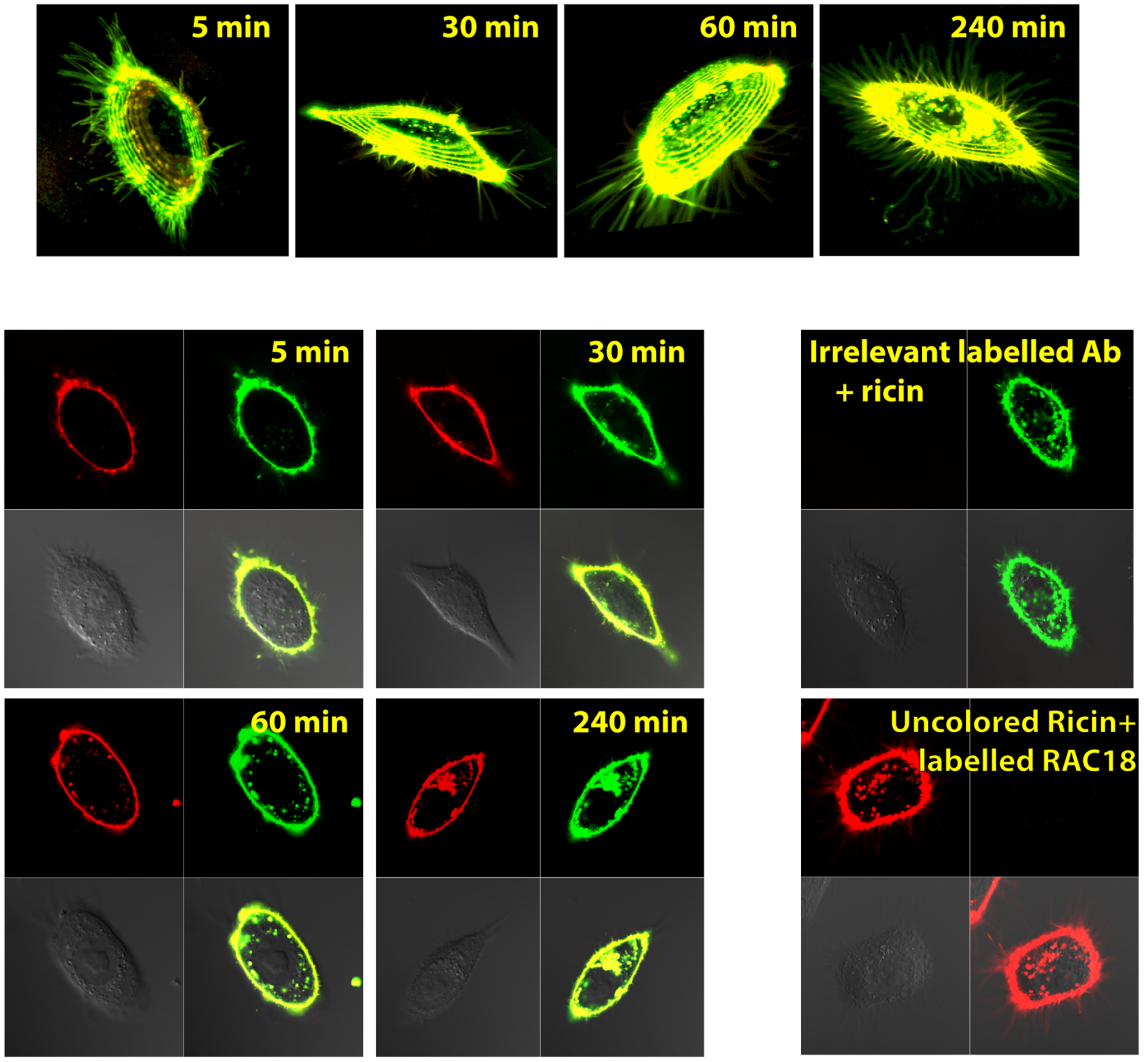
Colocalization of ricin and mAb RAC18 following internalization into cells. Live cells were incubated with labeled ricin (green) and RAC18 (red) and vertical stacks of confocal micrographs obtained at the indicated times. Colocalization of the two dyes appears yellow. At the top of the figure are the 3D representations of the merged fluorescent channels. At the bottom, a single plane from each stack shows the three channels imaged: red (Ab), green (ricin), and DIC, and then a merged image. Controls, which include labeled irrelevant Ab and unlabeled ricin with red-labeled RAC18, demonstrate that the colocalization is not an artifact of cross-channel readings. Panel C shows almost complete colocalization of Ab RAC18 (red) and ricin (green) 4 hr following ricin exposure. The micrographs are representative of >200 cells imaged in 5 distinct experiments.

For Ab to exert a prolonged effect on the trafficking of ricin, it must remain attached to ricin as it passes through different cell compartments. Because RAC18 Ab has been shown to directly neutralize ricin A chain’s enzymatic activity in a cell-free translation system [14], it is also possible that if the Ab remains attached to the ricin when it reaches the ribosome, it will inhibit the toxin’s N-glycosidase activity at its cellular site of action. To determine whether ricin and Ab remain colocalized, RAC18 and ricin were each labeled with different Alexa fluors, premixed, and added to cells. Images were obtained by fixing cells at different times after the addition of the immune complexes (figure 11). The ricin and Ab remain highly colocalized for at least 4 hr.

### Ab Protects Cells Even When Administered after Ricin has Entered the Cell

**Figure 12:**
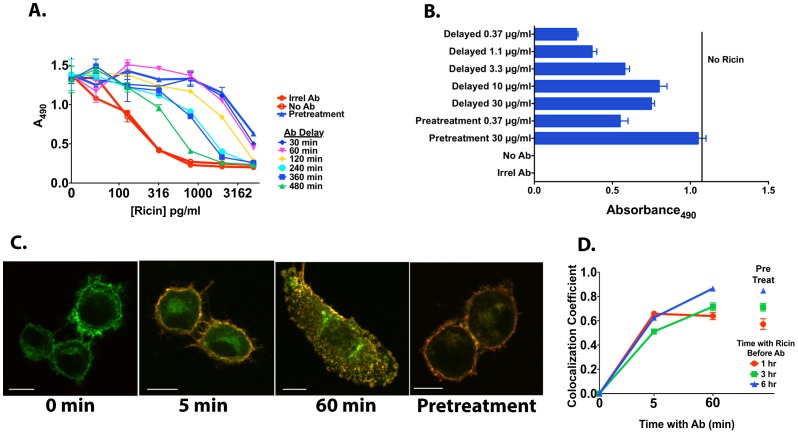
Ab administration may be delayed and still retain protective activity. A. H9 cells were cultured in triplicate in the presence of the indicated concentrations of ricin. RAC18 mAb (30 µg/ml) was either present prior to the ricin (pretreatment), or added after the indicated delay. 48hr later, an MTS dye reduction assay was performed. B. Same assay as in A, but with one concentration of ricin (1 ng/ml), and a fixed delay of 4 hr. Experiments demonstrating the protective effect of delayed ricin, similar to A and B, have been repeated >5 times. C. Confocal micrographs in which cells were incubated with labeled ricin (green) 3.6 µg/ml for 6 hr prior to the addition of the Ab (red) at 30 µg/ml. Times under micrographs indicate min after addition of Ab. Pretreatment had Ab present prior to ricin, and was incubated 6 hr. White bars in micrographs are 10 µm. D. Quantitative analysis of cells stained as described in panel C. ROIs were drawn to include all ricin associated with each cell. Ten cells were studied and the colocalization coefficient, the proportion of ricin bound to Ab, was determined for each (mean and SEM shown).

A corollary of the belief that Abs neutralize toxins extracellularly, is that once the cell has ingested ricin, it is too late for the Ab to exert its effect. On the other hand, if the primary site of neutralization is intracellular, then Ab may be effective even after ricin has entered the cell. We have demonstrated that ricin internalizes, distributes to critical cell compartments, and reaches a steady state within target cells by 60 min (figure 2). We therefore tested the effect of delaying Ab administration for up to 8 hr following exposure to ricin (figure 12). In the first experiment (figure 12A), different times of delay were tested in the presence of a constant concentration of Ab. We compared the degree of protection obtained to that observed when Ab was added prior to the ricin (pretreatment). Delaying Ab 30 or 60 min after ricin administration had no deleterious effect on cell survival compared to pretreatment. Ab efficacy decreased as the time of delay increased from 2 to 8 hr. But even when Ab was delayed by 8 hr, some protection was afforded from ricin toxicity in this 48 hr assay. These studies were performed with H9 cells, but identical results were obtained using HeLa cells. In the experiment shown in figure 12B, a dose-response of Ab protection was performed when Ab was added after a 4 hr delay. The results show that a ten-fold increase in Ab concentration was required if administered 4 hr after ricin exposure (compare delayed treatment with 3.3 µg/ml Ab to pretreatment with 0.37 µg/ml).

**Figure 13:**
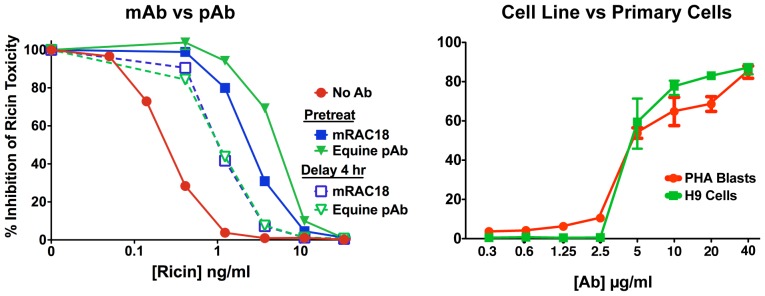
Measurements of cellular effects of Ab interactions with ricin are not influenced by source of Ab or type of cell tested. The ability of Ab to protect cells from ricin’s lethal effect was measured by MTS dye reduction at 48 hr (H9 cells) or 72 hr (PHA blasts). Results are shown as percent inhibition of ricin’s cytotoxic effect, mean and SEM of triplicate samples. Left. Equine anti-ricin Ab was compared to murine RAC18 for neutralization of cytotoxicity on H9 cells. Ab was added prior to ricin or 4 hr afterwards. Right. Protection of H9 cells or primary PHA-stimulated lymphoblasts by serial dilutions of mRAC18 in the presence of ricin, 3.3 ng/ml.

The events that occur with delayed Ab treatment are visualized in figure 12C. Cells were incubated with labeled ricin for 6 hr, resulting in abnormal morphology due to ricin toxicity and cellular response to the toxin. Images were taken immediately before, and at 5 min and 60 min following treatment with labeled Ab. The pretreatment control had Ab added prior to ricin, and was incubated for 6 hr. At 5 min the Ab had complexed with the ricin at the cell surface, but had not penetrated the cell. By 60 min the Ab had penetrated into the cell, yet there were still some areas where free ricin predominated. In the pretreatment control, Ab and ricin remain closely colocalized. Figure 12D quantifies what was visualized in 12C. The proportion of ricin that colocalized with Ab was calculated from 10 micrographs taken at each time point. It did not appear to make a major difference if the cells were incubated with ricin for 1, 3, or 6 hr. A significant amount of the Ab-ricin colocalization occurs by 5 min, likely at the cell surface. By 60 min, the proportion of ricin colocalizing with Ab was equal to that observed in the pretreatment controls. Results of experiments shown in figures 11 and 12 indicate that Ab likely neutralizes ricin toxin at intracellular sites. These data also provide an explanation for in vivo observations that indicate Ab administration may be delayed up to 24 hr post exposure and still exert a protective effect [16], [17]. All studies heretofore have been performed using monoclonal Abs and cell lines. In figure 13, we show that data obtained with polyclonal anti-ricin antibodies, and with primary human lymphoblasts yield similar data.

## Discussion

Vaccines directed against microbial toxins have had a profound impact upon human health, eliminating tetanus and diphtheria where immunization is regularly used. With increasing concern regarding bioterrorism, and with a deeper understanding of the role played by microbial toxins in human diseases, there is a renewed emphasis on developing vaccines and passive immunotherapies for toxin-mediated diseases, including anthrax [31], [32], [33], *C. difficile*-induced pseudomembranous colitis [34], botulism [35] and bacterial dysentery [13], [15], [36]. In seeking to understand how effective human vaccines function, there are very few facts upon which there is general agreement. One of these truisms is that protective anti-toxin immunity is mediated by Ab. Beyond that, our understanding of how antibodies actually function to protect cells is confused. We are taught that Ab functions by blocking the binding of toxin to cells, and this affects our thinking about how to design vaccines and target toxins with passive immunotherapy. Yet we also know that for some toxins, anti-A chain Abs neutralize toxic effects far better than anti-B chain Abs, even though anti-B chain Abs are those most likely to block binding of toxin to the cell. Anti-A chain Abs to ricin and its close functional relative, shiga toxin, are good examples of this phenomenon [13], [14]. The present studies ask how anti-A chain antibodies function to protect cells. The results indicate that Ab delays entry of toxin into the cell, influences intracellular routing of toxin to its site of action, remains attached to ricin during intracellular trafficking, and neutralizes A chain enzymatic activity inside the cell. Internal neutralization has also been suggested to occur with shiga toxin [15].

Ricin enters cells primarily through endocytosis of toxin bound to surface glycoproteins and glycolipids. When cells encounter ricin, we observe that a substantial proportion of the cell-associated toxin is internalized within 15 min, and plateaus in 40–60 min (figure 5), results that are substantially the same as observed in earlier studies using lower concentrations (100 ng/ml) of ^125^I-labeled ricin [27], [28]. To kill a cell, ricin must be transported in a retrograde fashion through the protein synthetic pathway, via the Golgi, ER, and finally the ribosome, where the A chain’s enzymatic activity catalytically removes adenine 4324 from the 18S rRNA, at the rate of 1500 ribosomes per min [37], [38]. Figures 5,8, and 9 show ricin has entered these cellular compartments within an hour, in accordance with ultrastructural studies performed previously by others [21], [22], [23], [24], [25], [26]. Ricin is extruded from the cell in ricin-coated, and ricin-containing vesicles, with blebbing likely playing a role (figure 5, videos S1 and S2). Sandvig and coworkers have previously demonstrated that 60–80% of the ricin that enters the cell is exocytosed within one hour [27], [28]. Although they don’t use the term blebbing, they do describe “recycling vesicles and tubules on the exocytic side”, “swarms of small vesicles throughout the cytoplasm”, “ruffling of the membrane”, and note that “ricin was seen in smaller vesicular structures, the nature of which was impossible to determine, although they may represent secretory vesicles” [21], [23]. Our studies provide a clear demonstration that blebbing occurs vigorously and early, suggesting that cellular irritation occurs long before apoptotic effects are observed (compare time scales in figures 5 and 6). The stimulus for blebbing could result from ricin’s ability to cross-link cellular glycoproteins and lipids. Blebbing may also serve to increase the exocytosis of ricin. Ricin is also removed by lysosomal degradation. It has been previously estimated that for every 10,000 ricin molecules entering the cell, only one reaches and cleaves ribosomes [39]. Cell death, primarily by apoptosis, takes 24 hrs or more (figure 6). Thus there may be a window, after exposure to this toxin, during which the cell may be rescued. A demonstration that toxic effects can be ameliorated even after the toxin has entered the cell is the recent use of inhibitors of retrograde transport to treat ricin poisoning [40].

In our studies, nAb first encounters toxin in the extracellular milieu, where a substantial amount of nAb and antigen binding occurs. Figure 8 confirms that Ab affinity/avidity is a strong correlate of neutralization, findings consistent with earlier studies with the same [14] and other [35], [41] mAbs. Both free and Ab-bound ricin attaches to cells, where complexes that include ricin, Ab, and cell-surface glycans are formed (figure 7). As more Ab binds to ricin, larger immune complexes develop. Entry of higher-order complexes containing Ab into cells is slowed, relative to complexes containing only ricin and cell-surface protein (figure 8). This may be caused by decreased lateral mobility of the larger complexes in the cell membrane (figure 10). Ironically, the presence of nAb results in an increased amount of cell-surface ricin, which is exactly the opposite of what we teach our students in basic immunology courses [2], [3], [4]. The delay of entry of ricin into the cell in turn delays entry of the toxin into the intracellular compartments it must traverse to reach its target (figure 9), allowing the cell more opportunity to rid itself of the toxin. Here we show that the Ab remains colocalized with ricin for hours in the cell (figure 11), and may thereby hinder translocation of ricin from ER to cytosol. In prior work we demonstrated that RAC18 was highly effective at neutralizing ricin’s enzymatic activity, in a cell-free, reticulocyte-lysate translation assay [14], raising the possibility that nAb inhibits the A chain’s enzymatic activity at its cellular site of action. To address this, we are performing fluorescent resonance energy transfer (FRET) to determine that the Ab is still bound to the ricin and not simply colocalized. It is also possible that simply by slowing the trafficking of the toxin through the cell, nAb shifts the balance from entry towards expulsion/degradation of the toxin. However it is the demonstration that Ab can mediate protection, even when added up to 8 hrs following ricin (figures 12 and 13), that most clearly shows that Ab can neutralize toxin intracellularly.

Intracellular function of Ab has been demonstrated previously. One of the earliest demonstrations was in hybridoma cells secreting anti-ricin Ab. The secreted Ab protected these cells from ricin by intercepting the toxin as it moved in a retrograde fashion through the protein synthetic pathway [42]. In other experiments, cells were protected from diphtheria toxin by intracellular introduction of anti-A chain Ab using RBC ghosts [43]. Anti-rotavirus VP6 mAbs, which do not neutralize extracellular virus, have been introduced into cells via lipid-mediated uptake. There mAb binds VP6, rendering the virus transcriptionally incompetent, and blocking virus replication [44]. Similarly the genetic expression of transfected Abs, so called intrabodies, has shown efficacy against endogenous [45] and exogenous [46] intracellular targets. Therefore, it should come as no surprise that if there is a physiological mechanism to allow entry of Abs into cells, that these Abs could then function in the intracellular milieu. Ab to adenovirus can enter cells attached to virions, and mediate virus degradation via TRIM21 [47]. Ab to intracellular bacteria can enter phagocytic cells via cell-surface FcR mediated function and target the bacteria to lysosomes [48]. In our experiments, it appears that Abs enter the cell by “hitchhiking” with the ricin as it is internalized. In the absence of antigen, Ab may enter cells through other routes, including fluid phase pinocytosis and FcR-dependent mechanisms.

Imaging live cells can introduce artifacts that may skew results. Repeated exposure of cells to light may induce phototoxicity. To avoid this, we have imaged cells only a single time in our studies of the effects of Ab on ricin internalization and intracellular routing. To visualize fluorescent ricin, we used concentrations of 3.6 µg/ml. Although this is the same concentration of ricin used in earlier studies of the intracellular routing of ricin [21], [23], [24], [25], [26], [28], [39], [49], we studied the kinetics of cell death when cells were exposed to ricin at 10 µg/ml or 10 ng/ml. We observed little difference in the kinetics of annexin V binding (figure 3), caspase cleavage (western blots, not shown), or inhibition of oxidative metabolism (MTS dye reduction, not shown) at these two concentrations. To avoid including ricin found in invaginations of the cell surface as having been internalized, we only used data from cell planes >1 µm from the top or bottom of the cell.

It seems likely that different toxins are best neutralized through different mechanisms, and that these mechanisms are tailored to the mode of action of the toxin. Both ricin and shiga toxins have N-glycosidase activity, cleaving the same ribosomal nucleic acid. Therefore it is not unexpected that both function intracellularly (this manuscript and reference [15]). Anthrax toxin forms a pore in the cell membrane, and the B chain, whose binding initiates the pore formation, is the major target of neutralizing Abs and has been rightly called “protective antigen” for many years. Diphtheria toxin targets transcription of RNA, an intracellular process. The importance of anti-B chain Abs in neutralization was initially highlighted [5], [6], but it is now well established that Abs to both A and B chains neutralize diphtheria toxin [9], [43], with many of the anti-A directly inhibiting the enzymatic function of the toxin. In the case of ricin, Abs to the B chain do have the ability to block binding of ricin to cells, and to protect cells from ricin, [14]; they just aren’t as efficient as anti-A chain Abs. In the case of active immunization, where poor Ab responses to B chain are typically obtained [14], [50], it is possible that the lectin properties of ricin B chain may alter and lessen the anti-B chain Ab response. We are currently studying the effects of anti-B chain mAbs on ricin internalization and intracellular trafficking.

These studies have implications for public health and biodefense. While not exceptionally lethal (LD_50_ 10–30 µg/kg), ricin’s easy availability, simple extraction, and chemical stability make it the “poor man’s” toxin of choice. Aerosol exposure in a small crowded space could produce symptoms and perhaps serious morbidity in those exposed. But, the likelihood of any one person being at risk for such an attack is extremely low. While a vaccine could have a place in a military setting if it were thought that the enemy could have ricin in its arsenal [51], [52], [53], the only realistic approach to protect civilian populations from such an attack is post-exposure treatment. Delay of specific diagnosis and obtaining antiserum could result in 12–24 hr delay in Ab administration following exposure. Thus our findings that Ab is protective even when administered well after the ricin has entered the cell, provide theoretical support for the potential efficacy of post-exposure therapy. Together with previous in vivo studies showing efficacy of delayed Ab treatment, the utility of post-exposure treatment for ricin exposure is supported.

## Supporting Information

Video S1 **Effects of ricin exposure, corresponding to** **figure 5A** **.** HeLa cells were stained with nuclei blue (Hoechst), ER and Golgi are red with Bodipy-brefeldin. Ricin (green) was added at 4 min. Individual cells were repeatedly imaged with a wide field water immersion objective. The time is indicated in min and sec. The cell under observation partially detaches at 10 minutes, and presents a side view thereafter.
(MP4)

Video S2 **Effects of ricin exposure, corresponding to** **figure 5B** **.** Performed as described for video S1.
(MP4)

Video S3 **Vertical (z) stacks of cells incubated with transferrin and ricin in the absence of Ab, corresponding to micrograph in** **figure 7** **.** Live HeLa cells were incubated with ricin (green) and transferrin (red) for ten minutes at 37°, in the absence of Ab. Vertical confocal sections were obtained at 0.6 µm intervals. Ricin and transferrin traffic through the cell in a similar fashion.
(MP4)

Video S4 **Vertical (z) stacks of cells incubated with transferrin and ricin in the presence of neutralizing Ab, corresponding to micrograph in** **figure 7** **.** Performed as described for video S3, but with the addition of neutralizing mAb RAC18 (10 µg/ml). Ricin accumulates at the cell surface, as transferrin freely enters.
(MP4)

Video S5 **Vertical (z) stacks of cells incubated with transferrin and ricin in the presence of neutralizing Ab, corresponding to micrograph in** **figure 7** **.** Performed as described for video S3, but with the addition of neutralizing mAb RAC18 (10 µg/ml). Ricin accumulates at the cell surface, as transferrin freely enters.
(MP4)

Video S6 **Vertical (z) stacks of cells incubated with transferrin and ricin in the presence of non-neutralizing Ab, corresponding to micrograph in** **figure 7** **.** Performed as described for video S3, but with the addition of non-neutralizing mAb RAC23 (10 µg/ml). Internalization of ricin is not affected by the addition of non-neutralizing Ab.
(MP4)

Video S7 **Time lapse micrographs showing the effect of neutralizing Ab on fluorescent recovery after photobleaching, corresponding to** **figure 10** **.** Live HeLa cells were incubated with Alexa 488-conjugated ricin. The region indicated by the red square was exposed to high intensity laser light, and then images obtained serially thereafter. Cells were incubated with 10 µg/ml of neutralizing mAb RAC18. The curves shown at the top right of figure 10 were obtained from these micrographs.
(MOV)

Video S8 **Time lapse micrographs showing fluorescent recovery after photobleaching in the absence of Ab, corresponding to** **figure 10** **.** Performed as in video S7, but in the absence of Ab. The curves shown at the top right of figure 10 were obtained from these micrographs.
(MOV)

Video S9 **Time lapse micrographs showing the effect of irrelevant Ab on fluorescent recovery after photobleaching, corresponding to** **figure 10** **.** Performed as in video S7, but in the presence of irrelevant Ab 924 (10 µg/ml). The curves shown at the top right of figure 10 were obtained from these micrographs.
(MOV)
